# Supplementary material for: Measuring the Diameter of Single-Wall Carbon Nanotubes Using AFM
Source: Nanomaterials (Basel). 2023 Jan 24;13(3):477. doi: 10.3390/nano13030477 (PMC9921789; doi:10.3390/nano13030477)
Supplement: Supplementary file 1 [file nanomaterials-13-00477-s001.zip › nanomaterials-2145078-supplementary.pdf]

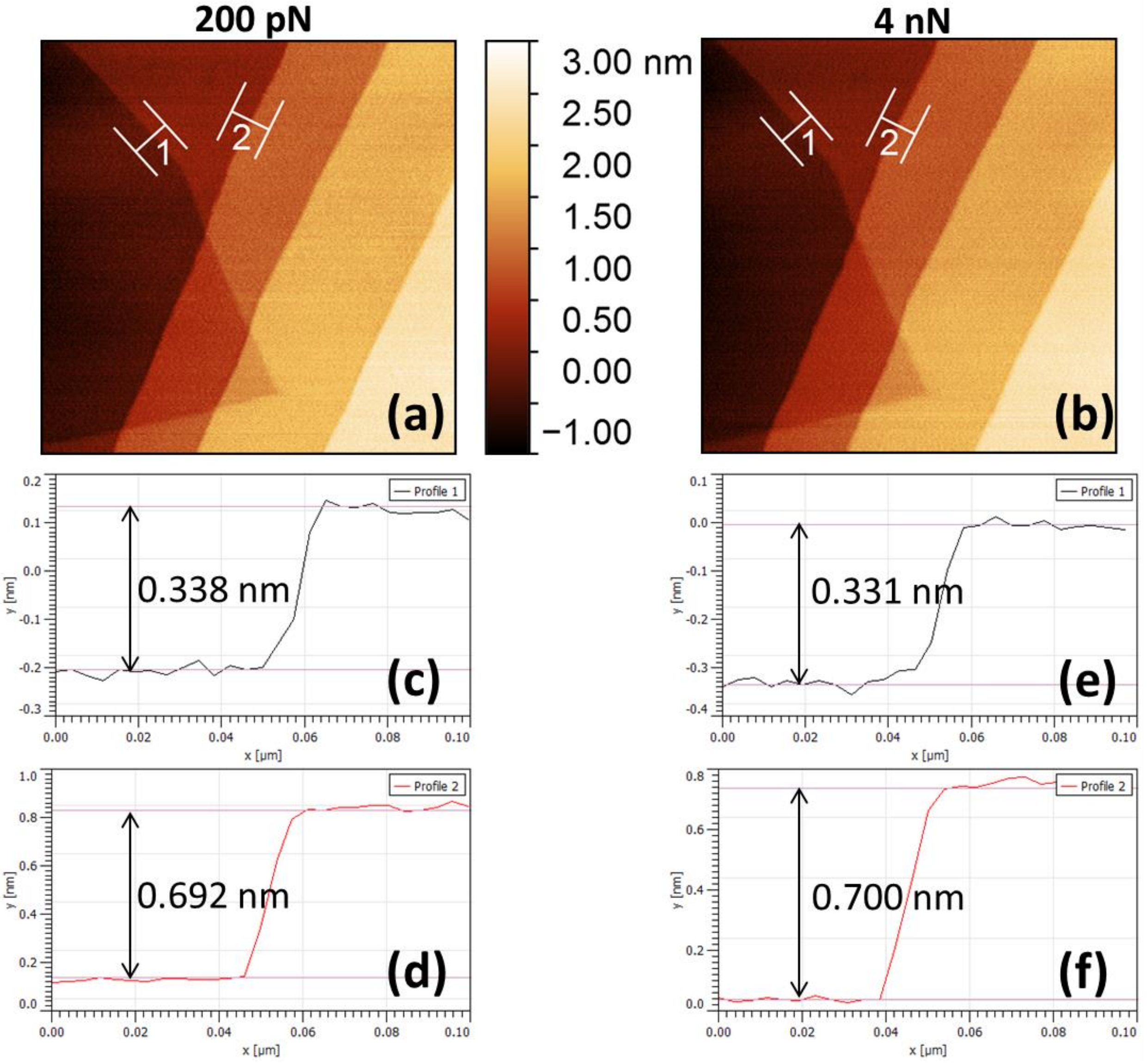

Figure S1. AFM images in (**a**) and (**b**), 1 µm^2 in size, show an HOPG area where several single and double atomic steps are visible; (**a**) was scanned with the peak force AFM feedback set to 200 pN, while (**b**) was scanned with it set to 4 nN; (**c**) and **(d**) correspond to cross-sections 1 and 2 in (**a**), while (**e**) and (**f**) correspond to the cross-sections in (**b**).

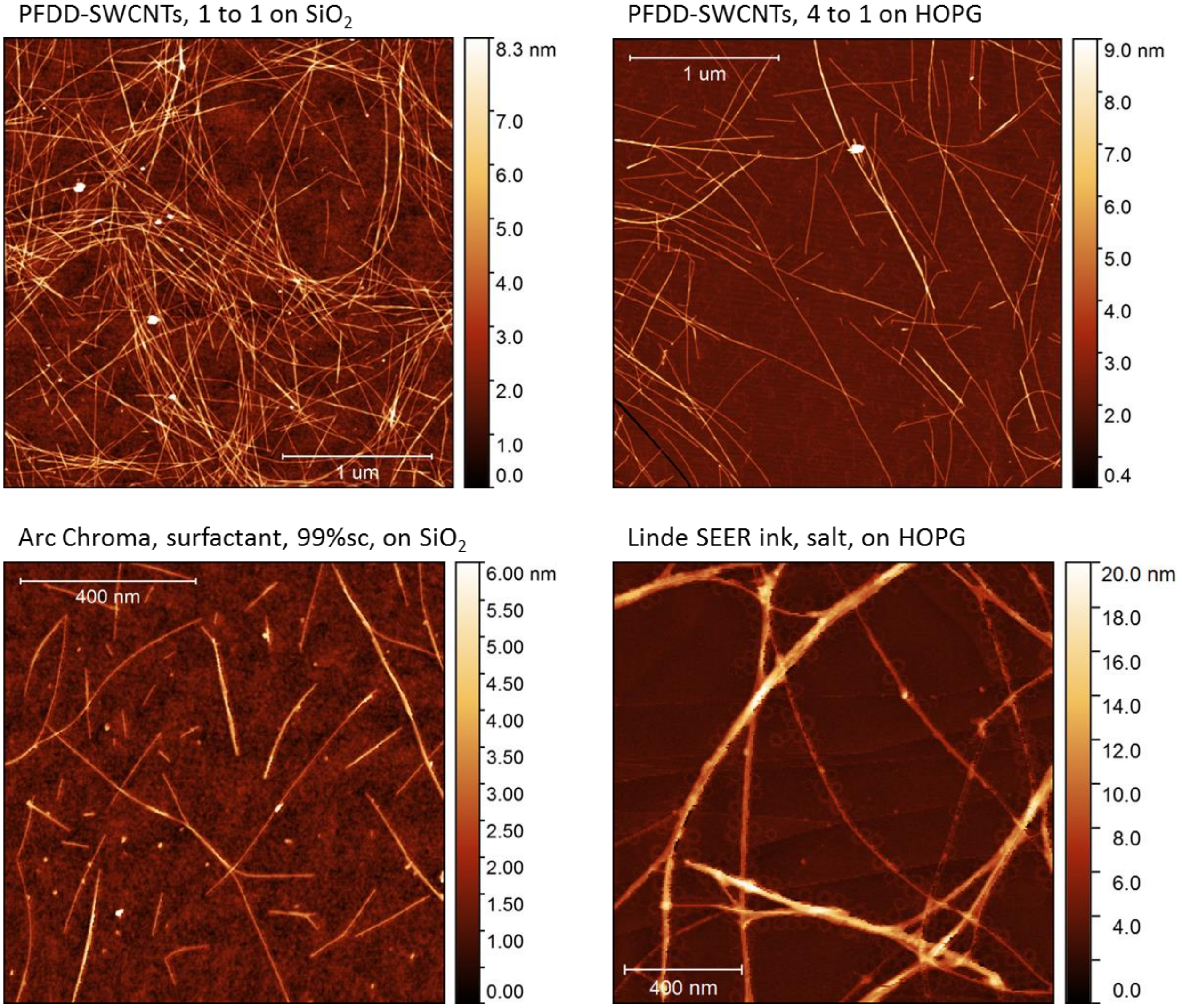

Figure S2. Representative AFM images are shown of networks made by dropcasting of four different commercially available SWCNT dispersions.

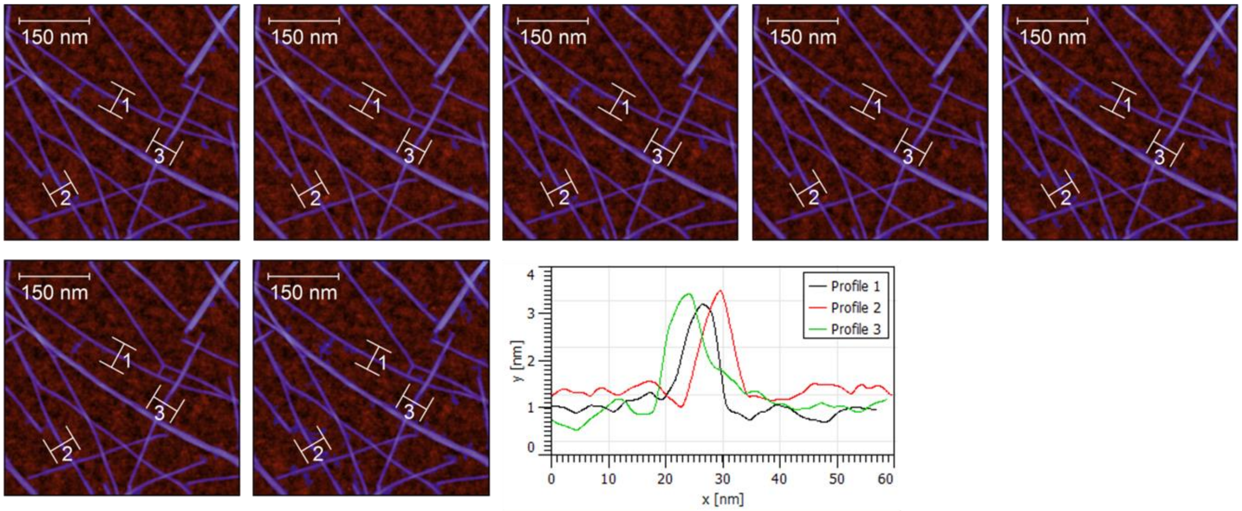

Scan #	Profile 1 height (nm)	Profile 2 height (nm)	Profile 3 height (nm)
Average (nm)	2.16	2.33	2.38
**Stan. Dev. (nm)**	**0.06**	**0.04**	**0.07**

Figure S3. We have taken the same image of a PFDD/SWCNT network on SiO_2 and have performed seven times our analysis method to extract diameters of several SWCNTs. In this example, three diameters were extracted using 30 pixel wide cross-sections as shown in the images. The analysis was performed on different days, not in a row, but following the same basic steps outlined in the article. This resulted in a variability of extracted diameters. The table in the bottom shows the average of seven measurements for each of the nanotubes, as well as the standard deviation. The standard deviation varies between 0.04 and 0.07 nm, which illustrates well the more generally observed range. Thus, our method allowed reducing the analysis uncertainty to below 0.1 nm.

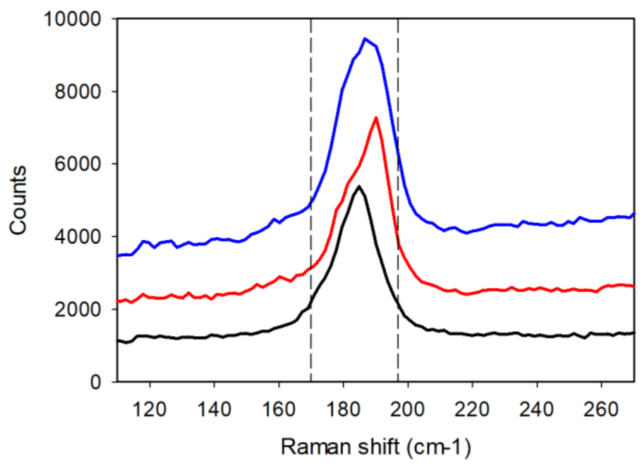

Figure S4. Raman spectra (514nm excitation) for three different spots on a sample of PFDD/SWCNTs on SiO_2 in the radial breathing mode (RBM) region. The dashed lines at 170cm^{-1} and 197cm^{-1} correspond to the Raman shift expected for RBMs of tubes with a diameter of 1.4nm and 1.2nm respectively.
